# Phosphorescent iridium(iii) complexes capable of imaging and distinguishing between exogenous and endogenous analytes in living cells†
†Electronic supplementary information (ESI) available: Synthesis, characterization, experimental information, and additional figures. See DOI: 10.1039/c8sc02984a


**DOI:** 10.1039/c8sc02984a

**Published:** 2018-08-03

**Authors:** Kenneth Yin Zhang, Taiwei Zhang, Huanjie Wei, Qi Wu, Shujuan Liu, Qiang Zhao, Wei Huang

**Affiliations:** a Key Laboratory for Organic Electronics and Information Displays and Jiangsu Key Laboratory for Biosensors , Institute of Advanced Materials (IAM) , Jiangsu National Synergetic Innovation Center for Advanced Materials (SICAM) , Nanjing University of Posts & Telecommunications , 9 Wenyuan Road , Nanjing 210023 , P. R. China . Email: iamqzhao@njupt.edu.cn ; Email: wei-huang@njtech.edu.cn; b Xi'an Institute of Flexible Electronics (XIFE) , Northwestern Polytechnical University (NPU) , 127 West Youyi Road , Xi'an 710072 , P. R. China

## Abstract

Many luminescent probes have been developed for intracellular imaging and sensing. During cellular luminescence sensing, it is difficult to distinguish species generated inside cells from those internalized from extracellular environments since they are chemically the same and lead to the same luminescence response of the probes. Considering that endogenous species usually give more information about the physiological and pathological parameters of the cells while internalized species often reflect the extracellular environmental conditions, we herein reported a series of cyclometalated iridium(iii) complexes as phosphorescent probes that are partially retained in the cell membrane during their cellular uptake. The utilization of the probes for sensing and distinguishing between exogenous and endogenous analytes has been demonstrated using hypoxia and hypochlorite as two examples of target analytes. The endogenous analytes lead to the luminescence response of the intracellular probes while the exogenous analytes are reported by the probes retained in the cell membrane during their internalization.

## Abstract 2

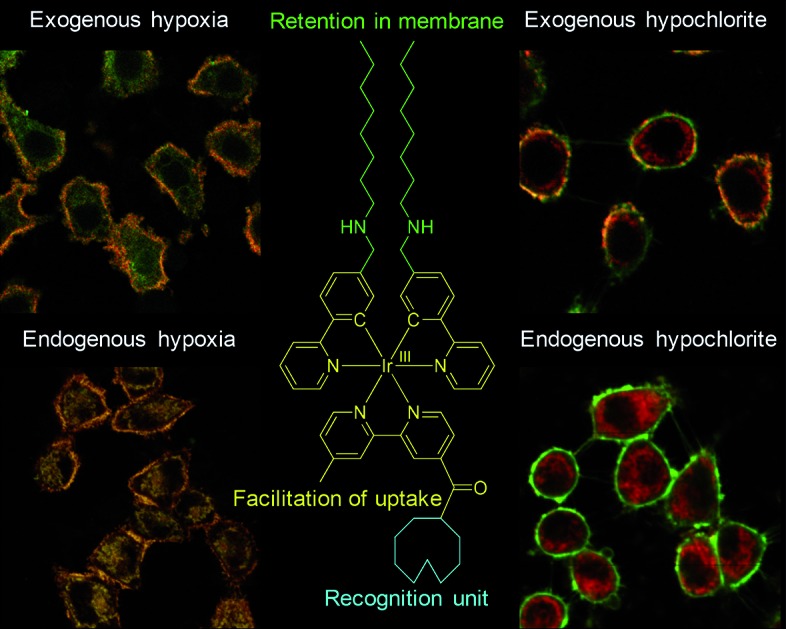
Phosphorescent iridium(iii) complexes bearing two carbon chains are able to distinguish between endogenous and exogenous analytes when serving as luminescent cellular probes.

## Introduction

Molecular probes showing a luminescence response toward specific analytes have been widely used for the detection of intracellular species related to physiological and pathological processes.^1^–^5^ The targets of interest mainly include metal cations involved in cellular processes,^6^–^8^ reactive oxygen/nitrogen species (RONS) that induce high oxidative stress,^9^–^11^ gasotransmitters that play roles in neurotransmission,^11^–^13^ enzymes that catalyze specific cellular reactions,^14^–^16^ characteristics of diseases such as pH values^16^–^18^ and hypoxia,^19^,^20^ *etc.* Many of the probes exhibit a sensitive response towards specific analytes and are used to determine their intracellular location and concentration *via* laser-scanning confocal microscopy, no matter whether the targets are produced inside the cells or internalized from extracellular environments. However, it is very difficult to distinguish between endogenous and exogenous species, because both of them lead to the same luminescence response of the probes. Since endogenously generated species usually give more information about the physiological and pathological conditions of the cells while internalized species often reflect the conditions of extracellular environments, it is of great importance to develop probes that are able to distinguish the origin of the analytes.

**Fig. 1:**
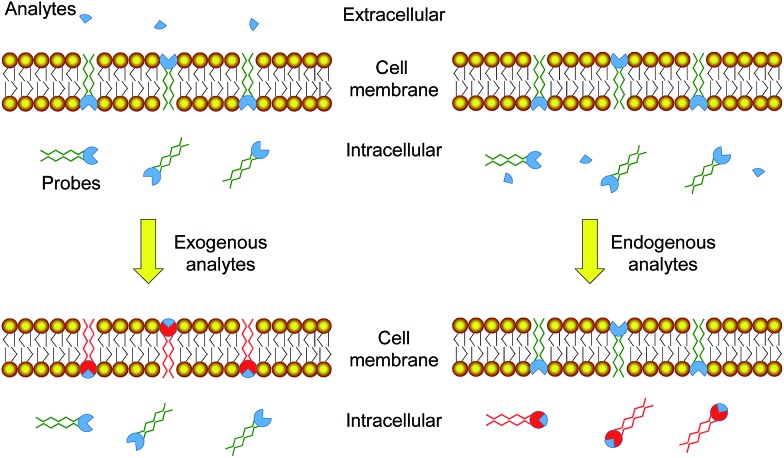
Strategy design of using luminescent probes that are partially retained in the cell membrane to distinguish between exogenous and endogenous analytes.

Endogenously generated and internalized species are chemically the same. The difference is that the internalized species must pass through the cell membrane while endogenously generated ones need not. Thus, we aim to develop luminescent probes that are partially retained in the cell membrane during their cellular uptake so that the internalized probes can report endogenously generated species while the retained probes are capable of monitoring the internalization of extracellular species (Fig. 1). Phosphorescent iridium(iii) polypyridine complexes are selected for this study because of their advantageous photophysical properties^21^–^25^ including intense phosphorescence and large Stokes shift. Their long luminescence lifetimes and high photostability facilitate photoluminescence lifetime imaging.^26^–^29^ Furthermore, the cytotoxicity^30^,^31^ and cellular distribution of iridium(iii) complexes are tunable *via* structural modification of the ligands. The utilization of iridium(iii) complexes to stain the cellular membrane,^32^ mitochondria,^31^ lysosomes,^33^ Golgi apparatus,^34^ nuclei,^35^ and nucleoli^36^ has been reported.

## Results and discussion

**Fig. 2:**
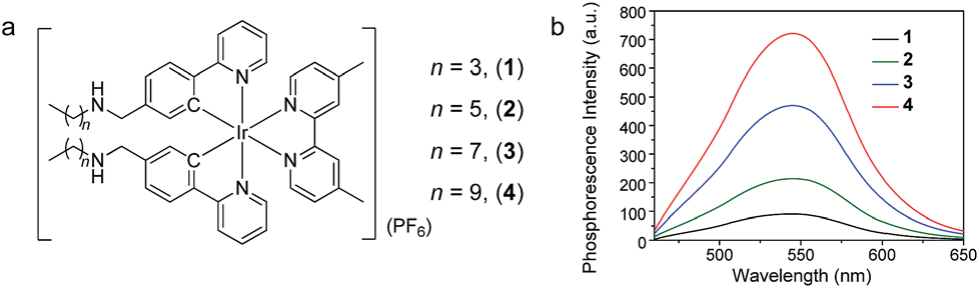
(a) Chemical structures of iridium(iii) complexes **1–4**. (b) Phosphorescence spectra of bilayer vesicles prepared from DSPC and complexes **1–4** in aerated PBS at 298 K upon photoexcitation at 405 nm.

In this work, phosphorescent iridium(iii) polypyridine complexes **1–4** (Fig. 2a) containing two lipophilic carbon chains of different lengths were designed and synthesized to study their cellular distribution, especially their retention in the cell membrane resulting from the lipophilic–lipophilic interaction with the lipid bilayer. The complexes have been characterized by ^1^H and ^13^C nuclear magnetic resonance (NMR), matrix-assisted laser desorption ionization time-of-flight (MALDI-TOF) mass spectrometry (MS), infrared (IR), and ultraviolet-visible (UV-Vis) absorption spectroscopy (see in the ESI†). Upon photoexcitation, all the complexes exhibited intense phosphorescence at about 545–550 nm with similar quantum yields of about 12–14% and lifetimes of about 375–398 ns in deaerated phosphate buffer saline (PBS, pH = 7.4)/DMSO (9 : 1, v/v), suggesting that the length of the carbon chain does not remarkably alter the photophysical properties of the complexes. To pre-evaluate the affinity of the complexes to cell membranes, bilayer vesicles were prepared from 1,2-distearoyl-*sn*-glycero-3-phosphocholine (DSPC) and the iridium(iii) complexes **1–4**, respectively according to the literature method.^37^ The luminescence spectra of the vesicle solutions in pure aqueous PBS were recorded and are shown in Fig. 2b. As the spectroscopic and luminescence properties of all the complexes are quite similar (Table S1†), the luminescence intensity of the vesicle solutions, to a certain extent, indicates the interaction of the complex with the bilayer vesicles. The luminescence intensity increased progressively with the length of the carbon chains, indicating that longer carbon chains in the complex structure strengthen the lipophilic–lipophilic interaction with the bilayer vesicles, probably facilitating the retention of the probes in the cell membrane.

**Fig. 3:**
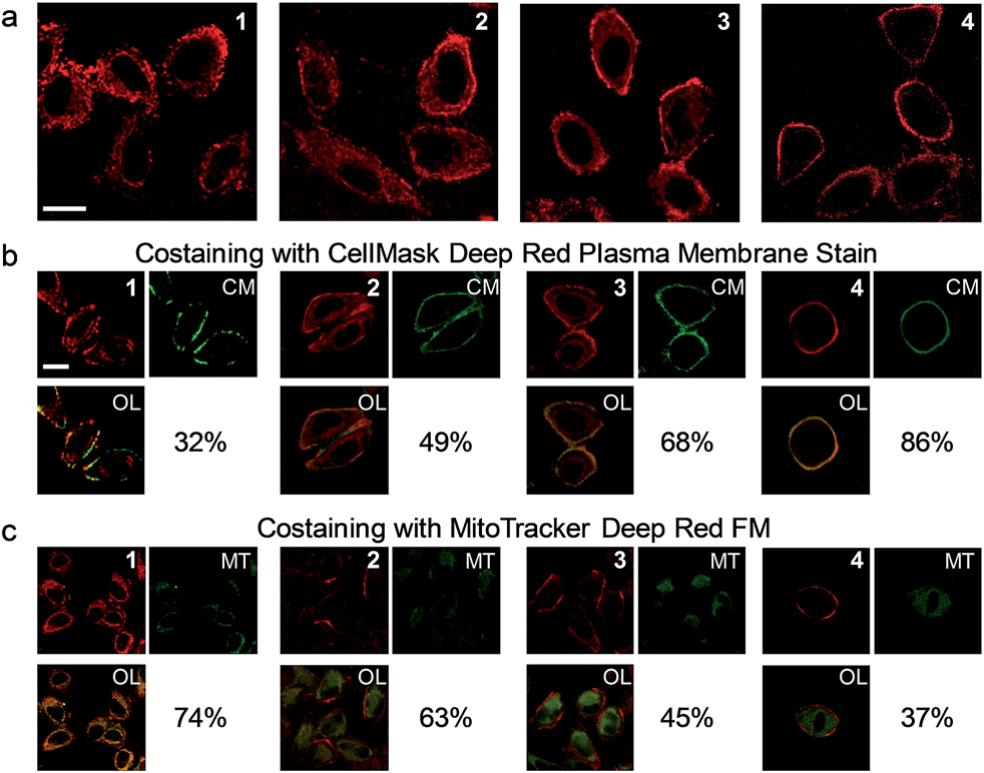
(a) Laser-scanning luminescence confocal microscopy images of living HeLa cells incubated with complexes **1–4** (5 μM, 20 min, 37 °C). (b) Images of the cells costained with complexes **1–4** and CellMask (CM). (c) Images of the cells costained with complexes **1–4** and MitoTracker (MT). OL: overlaid images. Percentage values: co-localization coefficients. The luminescence at 570 ± 50 and 670 ± 20 nm was collected for the complexes and fluorescent dyes, respectively. Scale bar: 20 μm.

The cell staining properties of the complexes have been studied *via* laser-scanning luminescence confocal microscopy. The MTT (3-[4,5-dimethylthiazol-2-yl]-2,5-diphenyltetrazolium bromide) assay revealed that HeLa cells maintained more than 80% viability after incubation with the complexes even at a high concentration of 100 μM for 24 h (Fig. S1†), indicative of the relatively low cytotoxicity of the complexes. Living HeLa cells incubated with the complexes (5 μM, 20 min, 37 °C) revealed intense cellular luminescence. Compared to many other iridium(iii) complexes that show efficient cellular internalization^21^–^25^ or specific organelle staining,^31^–^36^ complexes **1–4** were partially retained in the cell membrane. The internalized complexes were localized in the cytoplasm surrounding the cell nuclei (Fig. 3a). To determine the cellular distribution of the complexes, we performed costaining experiments involving a membrane staining dye, CellMask Deep Red Plasma Membrane Stain, and a mitochondria staining dye, MitoTracker Deep Red FM, respectively. Both dyes are excitable at 635 nm and emit at about 670 nm, which are well separated from the excitation (405 nm) and emission (550 nm) of the complexes. All the four complexes partially colocalized with CellMask and MitoTracker (Fig. 3b and c). The co-localization coefficients of complexes **1–4** with CellMask (32–86%) increased progressively with the length of the carbon chains, while a reverse trend was observed for the co-localization coefficients with MitoTracker (74–37%). These results reveal that the carbon chains partially inhibit the internalization of the complexes into living cells owing to the lipophilic–lipophilic interaction with the bilayer cell membrane and that the complexes with longer carbon chains exhibit a stronger affinity to the cell membrane. These results are in line with the luminescence spectra of complexes **1–4** in DSPC vesicles (Fig. 2b). Complex **3** was selected to develop luminescent probes for simultaneous intracellular and extracellular sensing and distinguishing between endogenous and exogenous analytes owing to its relatively equal distribution in the cell membrane and the cytoplasm (Fig. 3b and c). Prolonging the incubation time to as long as 6 h did not facilitate much the internalization of the complex into the cytoplasm; the co-localization coefficient of complex **3** with CellMask was slightly reduced to 0.65 (Fig. S2†), indicating that the retention of the complex in the cell membrane reached an equilibrium in less than 20 min and was stable for at least 6 h.

**Fig. 4:**
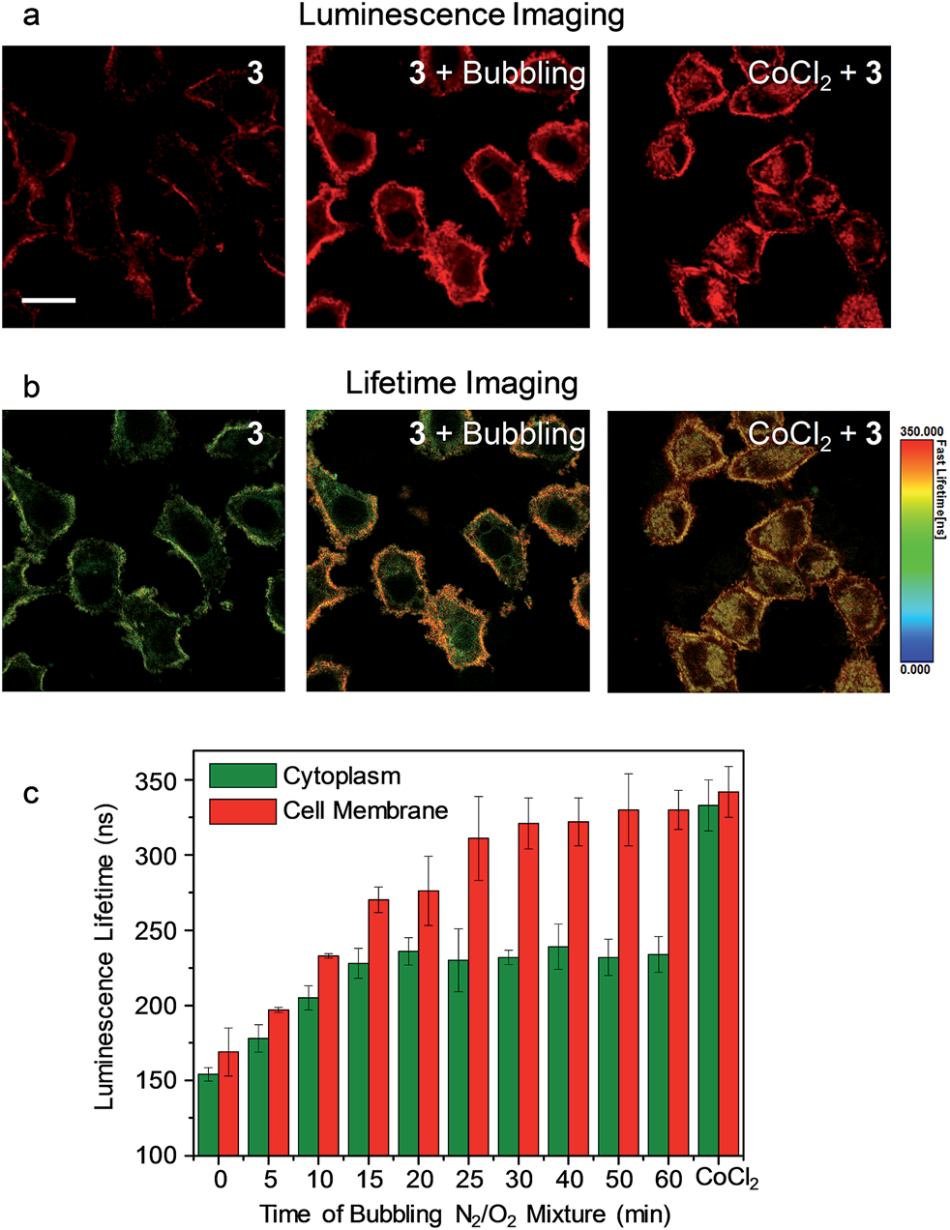
(a) Laser-scanning luminescence confocal microscopy and (b) photoluminescence lifetime imaging microscopy images of living HeLa cells incubated with complex **3** (5 μM, 20 min, 37 °C) before and after bubbling a gas mixture of 5% O_2_ and 95% N_2_ into the culture medium for 60 min and the cells pretreated with CoCl_2_ (100 μM, 2 h, 37 °C) and incubated with complex **3** (5 μM, 20 min, 37 °C). Scale bar: 20 μm. (c) Bar chart showing the luminescence lifetime values in the cytoplasm (green) and the cell membrane (red) of the HeLa cells incubated with complex **3** (5 μM, 20 min, 37 °C) during the bubbling gas mixture and after CoCl_2_ pretreatment. The error bars represent the standard deviations of ten lifetime values randomly obtained from independent cells.

As the phosphorescence of transition-metal complexes can be efficiently quenched by molecular oxygen *via* energy/electron transfer,^20^ we first demonstrated the utilization of complex **3** for cellular hypoxia sensing. Before cellular imaging, the phosphorescence spectra and lifetimes of complex **3** in DMSO/PBS (1 : 9, v/v) solution under an atmosphere containing different oxygen contents were recorded. Complex **3** exhibited phosphorescence enhancement by about 1.7 fold with lifetime elongation from 269 ns to 377 ns upon reduction of the oxygen content from 21% to 0 (Table S1†). The detailed results of the luminescence titration are shown in Fig. S3,† and the Stern–Volmer constant (*K*_SV_) was determined to be 0.027%^–1^. To eliminate the possible dynamic concentration variation of the complex in the cell membrane and the cytoplasm, the living cell imaging was performed *via* photoluminescence lifetime imaging microscopy (PLIM) owing to the independence of the lifetime values relative to the complex concentration. Living HeLa cells incubated with complex **3** (5 μM, 20 min, 37 °C) exhibited moderate phosphorescence from both the cytoplasm and the cell membrane (Fig. 4a) with similar lifetimes of about 154 ns and 169 ns, respectively (Fig. 4b). Bubbling a gas mixture of 5% O_2_ and 95% N_2_ into the culture medium with a flow rate of 5 mL min^–1^ gave rise to luminescence enhancement and lifetime elongation in both the cytoplasm and the cell membrane. Such a luminescence response reached an equilibrium after 30 min bubbling (Fig. 4c and S4†). Interestingly, exogenous hypoxia led to a more significant luminescence response in the cell membrane compared to that in the cytoplasm. Upon reaching equilibrium, the luminescence lifetime in the cell membrane was about 330 ns while that in the cytoplasm was about 100 ns shorter (Fig. 4b and c), indicating that the internalized complex **3** was less affected by exogenous hypoxia compared to the complex retained in the cell membrane. This is in accordance with our previous finding that the sensitivity of luminescent iridium(iii) complexes toward exogenous hypoxia was reduced upon their internalization into living cells.^20^ In this work, we also found that the complex retained in the cell membrane maintained high sensitivity toward exogenous hypoxia. To demonstrate the sensing of endogenous hypoxia by complex **3**, living HeLa cells were first treated with CoCl_2_ (100 μM, 2 h, 37 °C), which is a hypoxia inducer.^38^ Further incubation of the cells with complex **3** (5 μM, 20 min, 37 °C) led to intense luminescence from both the cytoplasm and the cell membrane (Fig. 4a). Compared to the CoCl_2_-untreated cells, the luminescence was much brighter and the lifetimes were much longer, which were determined to be about 333 ns and 342 ns in the cytoplasm and the cell membrane, respectively (Fig. 4b and c). Since the luminescence lifetime of complex **3** was hardly affected by CoCl_2_ in aqueous PBS buffer (Table S1†), the lifetime elongation has been ascribed to the intracellular hypoxia induced by CoCl_2_. According to the luminescence lifetime values obtained from the PLIM images, the complex retained in the cell membrane exhibited a similar response toward both exogenous and endogenous hypoxia. In contrast to the reduced sensitivity of the internalized complex toward exogenous hypoxia, it exhibited much more significant lifetime elongation in response to endogenous hypoxia (Fig. 4).

**Fig. 5:**
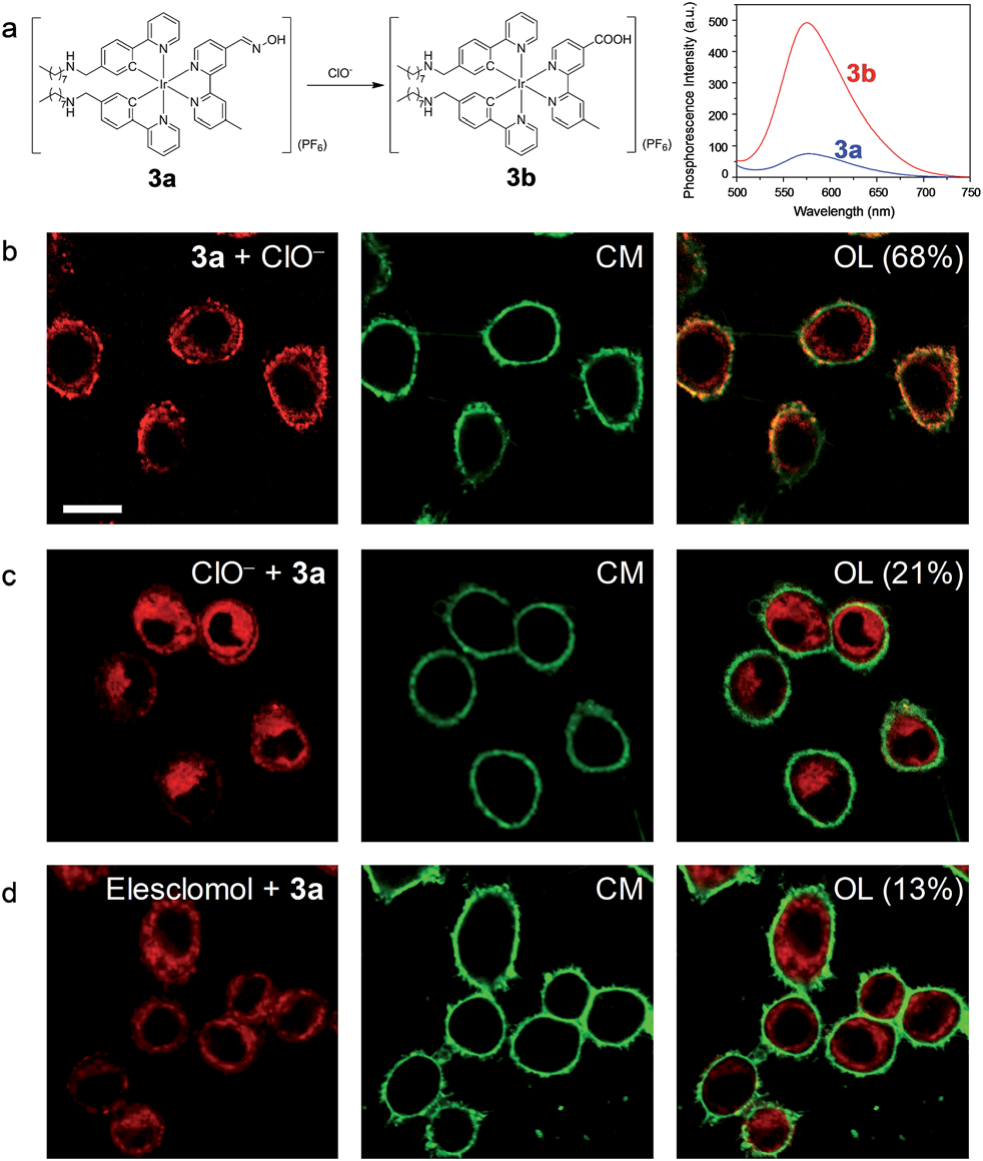
(a) Chemical structure of complex **3a** and the structural and spectral (in CH_3_OH) responses toward hypochlorite. (b) Images of living HeLa cells incubated with complex **3a** (5 μM, 20 min, 37 °C) followed by treatment with NaClO (25 μM, 20 min, 37 °C) and costaining with CellMask (CM). (c) Images of living HeLa cells preincubated with NaClO (25 μM, 20 min, 37 °C), washed with PBS three times, incubated with complex **3a** (5 μM, 20 min, 37 °C) and costained with CellMask. (d) Laser-scanning luminescence confocal microscopy images of living HeLa cells treated with elesclomol (125 nM, 2 h, 37 °C) followed by incubation with complex **3a** (5 μM, 20 min, 37 °C) and costaining with CellMask. OL: overlaid images. Percentage values: co-localization coefficients. Scale bar: 20 μm.

In another demonstration, an aldoxime group was incorporated into the diimine ligand of complex **3**, affording complex **3a** as a phosphorogenic probe for hypochlorite. Complex **3a** was weakly emissive due to the quenching by the isomerisation of the aldoxime group.^27^ In the presence of hypochlorite, the aldoxime group was converted to a carboxyl group, yielding complex **3b** (Fig. 5a) and resulting in luminescence enhancement (Fig. 5a and S5†).^27^ The phosphorogenic response of complex **3a** toward hypochlorite was in preference to other common biological anions and RONS (Fig. S6†). The MTT assay confirmed the good biocompatibility of complex **3a** (Fig. S7†). Living HeLa cells incubated with complex **3a** (5 μM, 20 min, 37 °C) did not reveal noticeable luminescence due to the weak emission of the complex (Fig. S8†). Further incubation of the cells with sodium hypochlorite as an exogenous hypochlorite source led to remarkable luminescence turn-on in the cell membrane (Fig. 5b), indicating that the complex retained in the cell membrane was oxidized to the carboxyl analogue **3b** by the exogenous hypochlorite during its internalization. In response to the internalized hypochlorite, the cytoplasm also revealed less intense luminescence compared to the cell membrane. A fairly high co-localization coefficient of 68% with CellMask was obtained. In sharp contrast, intense luminescence was observed in the perinuclear region with the cell membrane being weakly emissive (Fig. 5c), revealing a low co-localization coefficient of 21% with CellMask, when the cells were preloaded with sodium hypochlorite followed by washing with PBS and incubation with complex **3a**. This is because the internalization of hypochlorite had finished when the cells were incubated with complex **3a**. In a parallel experiment demonstrating the detection of endogenous hypochlorite, living HeLa cells were pretreated with elesclomol (125 nM, 2 h, 37 °C), which is an anticancer drug that induces oxidative stress by triggering production of RONS including hypochlorite.^39^ Incubation of the cells with complex **3a** resulted in bright luminescence in the perinuclear region with the cell membrane being non-emissive (Fig. 5d). The co-localization coefficient with CellMask was as low as 13%. To exclude the possible damage by hypochlorite or elesclomol to the cell membrane, we incubated the hypochlorite- and elesclomol-treated cells with complex **3**. Both the cytoplasm and the cell membrane were brightly emissive (Fig. S9†), ensuring the integrity of the cell membrane. These results suggested that the endogenous hypochlorite selectively oxidized the internalized complex **3a**, since the internalization of hypochlorite was not required.

## Conclusions

To conclude, we have developed a series of phosphorescent iridium(iii) complexes that stain both the cytoplasm and the cell membrane. The amounts of the complex retained in the cell membrane can be easily tuned by shortening or elongating the carbon chains in the complex structure. Incorporation of recognition units into the new luminescent probes not only allow to sense exogenous and endogenous analytes but also enable to distinguish them from each other. Since endogenous species usually reflect physiological and pathological parameters of the cells while exogenous species are more related to extracellular environmental conditions, these probes are of great importance and helpfulness in improving the accuracy and precision in disease diagnosis. However, these probes cannot be used for distinguishing when the endogenous analytes are produced in the cell membrane or can undergo exocytosis through the cell membrane.

## Conflicts of interest

There are no conflicts to declare.

## Supplementary Material

Supplementary information
