# Supplementary material for: Phosphorescent iridium(iii) complexes capable of imaging and distinguishing between exogenous and endogenous analytes in living cells
Source: Chem Sci. 2018 Aug 3;9(36):7236–40. doi: 10.1039/c8sc02984a (PMC6148462; doi:10.1039/c8sc02984a)

Electronic Supplementary Material (ESI) for Chemical Science.
This journal is © The Royal Society of Chemistry 2018

Supporting Information

Phosphorescent Iridium(III) Complexes Capable of Imaging and Distinguishing between Exogenous and Endogenous Analytes in Living Cells

Kenneth Yin Zhang,[a] Taiwei Zhang,[a] Huanjie Wei,[a] Qi Wu,[a] Shujuan Liu,[a] Qiang Zhao,*[a] and Wei Huang*[a,b]

[a] Key Laboratory for Organic Electronics and Information Displays and Jiangsu Key Laboratory for Biosensors, Institute of Advanced Materials (IAM), Jiangsu National Synergetic Innovation Center for Advanced Materials (SICAM), Nanjing University of Posts & Telecommunications, 9 Wenyuan Road, Nanjing 210023, P. R. China

[b] Xi'an Institute of Flexible Electronics (XIFE), Northwestern Polytechnical University (NPU), 127 West Youyi Road, Xi'an 710072, P. R. China

General experimental information. All solvents were of analytical grade and purified according to standard procedures (W. L. F. Armarego, C. L. L. Chai, *Purification of Laboratory Chemicals*, 6th ed., Elsevier, Oxford, **2009**). All chemicals used were purchased from J&K China Chemical Ltd. unless stated. ^{1}H NMR and ^{13}C NMR spectra were recorded on a Bruker ACF400 spectrometer at 298 K using deuterated solvents. Chemical shifts (δ, ppm) were reported relative to tetramethylsilane (TMS). Mass spectra were recorded on a Bruker autoflex matrix-assisted laser desorption ionization time-of-flight (MALDI-TOF) mass spectrometer (MS). Infrared spectra were recorded on a Perkin-Elmer 1600 series FT-IR spectrophotometer. UV-Vis absorption spectra were recorded on a UV-1700 Shimadzu UV-Vis spectrophotometer. Photoluminescence spectra and emission lifetimes were measured on an Edinburgh FL 920 spectrophotometer. ROS and RNS are prepared and quantified according to the literature (B. Wang, P. Li, F. Yu, P. Song, X. Sun, S. Yang, Z. Lou, K. Han, *Chem. Commun.* **2013**, *49*, 1014-1016; S.-R. Liu, S.-P. Wu, *Org. Lett.* **2013**, *15*, 878-881; A. M. Held, D. J. Halko, J. K. Hurst, *J. Am. Chem. Soc.* **1978**, *100*, 5732-5740; J. W. Reed, H. H. Ho, W. L. Jolly, *J. Am. Chem. Soc.* **1974**, *96*, 1248-1249; K. Chen, J. W. Bats, M. Schmittel, *Inorg. Chem.* **2013**, *52*, 12863-12865). The cytotoxic effect of all the complexes toward HeLa cells was studied using the 3-(4,5-dimethyl-2-thiazolyl)-2,5-diphenyltetrazolium bromide (MTT) assay (T. Mosmann, *J. Immunol. Methods* **1983**, *65*, 55-63). Luminescence microscopy imaging was carried out on an Olympus IX81 laser-scanning confocal microscope. The co-localization coefficients were calculated by ImageJ (Version 1.4.3.67). The FLIM setup is integrated with the same Olympus IX81 laser scanning confocal microscope. The lifetime values were calculated with professional software provided by PicoQuant Company.

Synthesis and characterization.

$[Ir_2(ppy\text{-}CH_2NH\text{-}C_nH_{2n+1})_4Cl_2]$ (n = 4, 6, 8, 10).

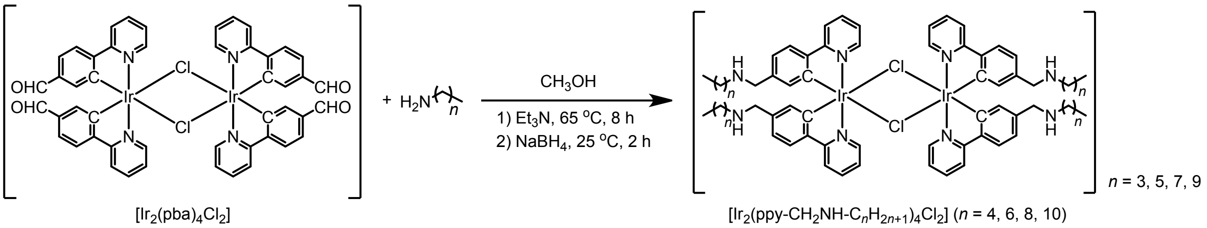

A mixture of $[Ir_2(pba)_4Cl_2]$ (K. K.-W. Lo, C.-K. Chung, N. Zhu, *Chem. Eur. J.* **2003**, *9*, 475-483) (296 mg, 0.25 mmol) and corresponding amine (1.5 mmol) in methanol (10 mL) containing triethylamine (500 μL) was refluxed under an inert atmosphere of nitrogen in the dark for 8 h. After the solution was cooled to room temperature, solid $NaBH_4$ (304 mg, 8 mmol) was added. The solution was stirred at room temperature for 2 h and then evaporated to dryness to give a yellow solid. The solid was dissolved in CH_2Cl_2 (50 mL) and the solution was washed with distilled water (20 mL × 3). The organic layer was collected, dried over anhydrous $MgSO_4$, and evaporated to dryness to give a yellow solid. The crude product was purified by column chromatography on silica gel. The desired product was eluted with CH_2Cl_2/methanol (50:1, v/v) and isolated as yellow solids.

$[Ir(ppy\text{-}CH_2NH\text{-}C_nH_{2n+1})_2(Me_2bpy)](PF_6)$ (n = 4, 6, 8, 10) (Complexes 1 – 4).

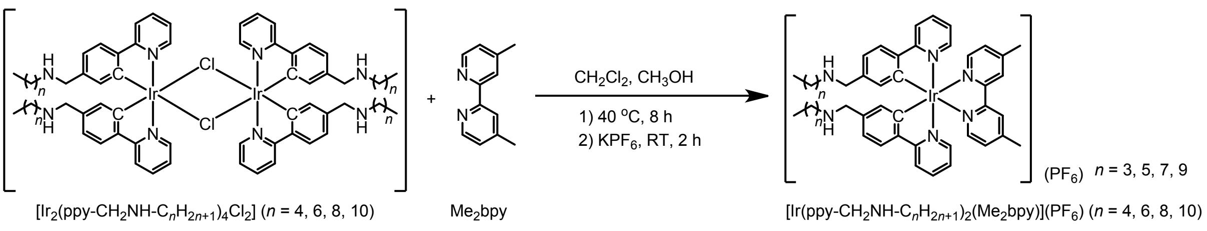

A mixture of $[Ir_2(ppy\text{-}CH_2NH\text{-}C_nH_{2n+1})_4Cl_2]$ (0.15 mmol) and Me_2bpy (0.35 mmol) in CH_3OH/CH_2Cl_2 (15 mL, 1:2, v:v) was refluxed under an inert atmosphere of nitrogen in the dark for 8 h. The solution was then cooled to room temperature and KPF_6 (276 mg, 1.5 mmol) was added. After stirring at room temperature for 2 h, the mixture was evaporated to dryness. The crude product was purified by column chromatography on silica gel. The desired product

was eluted with CH_2Cl_2/methanol (25:1, v/v) and isolated as yellow solids.

Complex 1. Complex **1** was recrystallized from CH_2Cl_2/diethyl ether and obtained as yellow crystals in 45% yield. ^{1}H NMR (400 MHz, $CDCl_3$, 25°C, TMS): δ = 8.40 (s, 2H), 7.89 (d, J = 7.8 Hz, 2H), 7.82 – 7.73 (m, 4H), 7.64 (d, J = 8.0 Hz, 2H), 7.54 (d, J = 5.7 Hz, 2H), 7.20 (d, J = 5.9 Hz, 2H), 7.07 – 6.99 (m, 4H), 6.20 (s, 2H), 3.60 – 3.48 (m, 4H), 2.59 (s, 6H), 2.56 – 2.51 (m, 4H), 1.45 – 1.25 (m, 8H), 0.89 ppm (t, J = 7.3 Hz, 6H); ^{13}C NMR (100 MHz, $CDCl_3$, 25°C, TMS): δ = 167.68, 155.45, 152.25, 150.93, 149.52, 148.55, 142.85, 142.28, 137.89, 131.12, 128.76, 125.92, 124.76, 122.99, 122.31, 119.31, 53.90, 49.14, 32.06, 21.36, 20.45, 14.04 ppm; IR (KBr) = 3438 (N–H), 844 cm^{-1} (PF_6^-); UV/Vis (DMSO): λ_{max} (ε / dm^{3}mol^{-1}cm^{-1}) = 416 (3855), 312 nm (24820); MALDI-TOF MS: m/z = 855 [M$^+$].

Complex 2. Complex **2** was recrystallized from CH_2Cl_2/diethyl ether and obtained as yellow crystals in 48% yield. ^{1}H NMR (400 MHz, $CDCl_3$, 25°C, TMS): δ = 8.46 (s, 2H), 7.87 (d, J = 8.2 Hz, 2H), 7.81 – 7.73 (m, 4H), 7.63 (d, J = 8.1 Hz, 2H), 7.52 (d, J = 5.7 Hz, 2H), 7.19 (d, J = 5.4 Hz, 2H), 7.06 – 6.98 (m, 4H), 6.19 (s, 2H), 3.64 – 3.47 (m, 4H), 2.59 (s, 6H), 2.52 – 2.47 (m, 4H), 1.46 – 1.38 (m, 4H), 1.29 – 1.24 (m, 12H), 0.88 ppm (t, J = 7.3 Hz, 6H); ^{13}C NMR (100 MHz, $CDCl_3$, 25°C, TMS): δ = 167.53, 155.40, 152.22, 150.93, 149.62, 148.62, 142.54, 137.95, 131.33, 128.83, 125.80, 124.80, 123.14, 122.47, 53.67, 49.40, 31.72, 29.54, 26.97, 22.63, 21.37, 14.09 ppm; IR (KBr) = 3435 (N–H), 846 cm^{-1} (PF_6^-); UV/Vis (DMSO): λ_{max} (ε / dm^{3}mol^{-1}cm^{-1}) = 418 (4845), 312 nm (32205); MALDI-TOF MS: m/z = 911 [M$^+$].

Complex 3. Complex **3** was recrystallized from CH_2Cl_2/diethyl ether and obtained as yellow crystals in 52% yield. ^{1}H NMR (400 MHz, $CDCl_3$, 25°C, TMS): δ = 8.27 (s, 2H), 7.80 (d, J = 5.5 Hz, 2H), 7.76 – 7.67 (m, 4H), 7.56 – 7.49 (m, 4H), 7.21 (d, J = 5.5 Hz, 2H), 7.04 – 6.98 (m, 4H), 6.23 (s, 2H), 3.65 – 3.45 (m, 4H), 2.72 – 2.59 (m, 4H), 2.51 (s, 6H), 1.63 – 1.46 (m, 4H), 1.27 – 1.11 (m, 20H), 0.84 ppm (t, J = 6.8 Hz, 6H); ^{13}C NMR (100 MHz, $CDCl_3$, 25°C, TMS): δ = 166.90, 155.16, 151.93, 150.88, 150.16, 148.88, 138.25, 131.99, 129.22, 127.74,

125.20, 124.99, 123.8, 123.18, 119.79, 58.46, 31.91, 29.58, 29.34, 26.97, 22.69, 21.56, 21.38, 18.44, 14.14 ppm; IR (KBr) = 3424 (N–H), 843 cm^{-1} (PF_6^-); UV/Vis (DMSO): λ_{max} (ε / $dm^3mol^{-1}cm^{-1}$) = 416 (3600), 310 nm (21360); MALDI-TOF MS: m/z = 967 [M^+].

Complex 4. Complex **4** was recrystallized from CH_2Cl_2/diethyl ether and obtained as yellow crystals in 44% yield. ^{1}H NMR (400 MHz, $CDCl_3$, 25°C, TMS): δ = 8.32 (s, 2H), 8.24 (d, J = 5.5 Hz, 2H), 7.79 – 7.74 (m, 4H), 7.60 (d, J = 6.5 Hz, 2H), 7.54 (d, J = 5.3 Hz, 2H), 7.22 (d, J = 5.0 Hz, 2H), 7.10 – 6.98 (m, 4H), 6.18 (s, 2H), 3.83 – 3.63 (m, 4H), 2.56 (s, 6H), 2.53 – 2.51 (m, 4H), 1.51 – 1.41 (m, 4H), 1.27 – 1.24 (m, 28H), 0.88 ppm (t, J = 6.8 Hz, 6H); ^{13}C NMR (100 MHz, $CDCl_3$, 25°C, TMS): δ = 166.90, 155.16, 151.93, 150.88, 150.16, 148.88, 138.25, 131.99, 129.22, 127.74, 125.20, 124.99, 123.81, 123.18, 119.79, 58.46, 31.91, 29.58, 29.34, 26.97, 22.69, 21.56, 21.38, 18.44, 14.14 ppm; IR (KBr) = 3437 (N–H), 847 cm^{-1} (PF_6^-); UV/Vis (DMSO): λ_{max} (ε / $dm^3mol^{-1}cm^{-1}$) = 420 (3160), 312 nm (25215); MALDI-TOF MS: m/z = 1023 [M^+].

Complex 3a. The procedure was similar to that for the preparation of complex **3**, except that the aldoxime-modified diimine ligand (N. Zhao, Y.-H. Wu, R.-M. Wang, L.-X. Shi, Z.-N. Chen, *Analyst*, **2011**, *136*, 2277-2282) was used instead of Me_2bpy. Complex **3a** was recrystallized from CH_2Cl_2/diethyl ether and obtained as orange crystals in 38% yield. ^{1}H NMR (400 MHz, $CDCl_3$, 25°C, TMS): δ = 9.13 (s, 1H), 8.72 (s, 1H), 8.19 (s, 1H), 7.92 – 7.85 (m, 2H), 7.84 – 7.80 (m, 2H), 7.78 – 7.72 (m, 3H), 7.67 – 7.61 (m, 2H), 7.56 – 7.44 (m, 2H), 7.34 – 7.19 (m, 2H), 7.07 – 6.94 (m, 4H), 6.20 (d, J = 10.9 Hz, 2H), 3.62 – 3.43 (m, 4H), 2.59 (s, 3H), 2.53 – 2.43 (m, 4H), 1.47 – 1.36 (m, 4H), 1.32 – 1.15 (m, 20H), 0.87 ppm (t, J = 7.1 Hz, 6H); ^{13}C NMR (100 MHz, $CDCl_3$, 25°C, TMS): δ = 167.91, 156.63, 155.03, 152.42, 150.17, 149.48, 148.49, 146.60, 145.77, 143.31, 141.02, 140.98, 137.85, 131.74, 129.05, 126.19, 124.58, 124.22, 122.76, 119.09, 35.97, 31.94, 30.80, 29.60, 29.45, 29.35, 29.22, 22.71, 21.35, 14.16 ppm; IR (KBr) = 3432 (N–H), 847 cm^{-1} (PF_6^-); UV/Vis (DMSO): λ_{max}

(ε / $dm^3mol^{-1}cm^{-1}$) = 420 (9928), 381 (22835), 325 nm (52930); MALDI-TOF MS: m/z = 996 [M^+].

Preparation of DSPC vesicles. 1,2-Distearoyl-*sn*-glycero-3-phosphocholine (DSPC) (1.99 mg, 2.5 μmol) in $CHCl_3$ (300 μL) was added to a solution of the complexes **1** – **4** (1 mg) in $CHCl_3$ (250 μL) in a glass test tube, respectively. The solvent was evaporated under a stream of purified dry nitrogen. PBS (pH 7.4, 1 mL) was added to the dried lipid film. The liquid suspension was then sonicated at 37 °C for 2 h. The solid residue was removed by filtration. The filtrate was diluted 10 times with PBS for measurement of luminescence spectra.

Table S1. Spectroscopic and photophysical data for complexes **1 – 4** and **3a**.

Complex	λ_{abs} / nm (log ε)^a	λ_{em} / nm^b	τ / ns^b	τ / ns^c	Φ^c
**1**	312 sh (4.39), 416 sh (3.59)	547	230	398	0.14
**2**	312 sh (4.51), 418 sh (3.69)	545	248	375	0.12
**3**	310 sh (4.33), 416 sh (3.56)	548	269^d	377	0.13
**4**	312 sh (4.40), 420 sh (3.50)	550	250	381	0.12
**3a**	325 sh (4.72), 381 sh (4.36), 420 sh (4.00)	575	^{e}	^{e}	<0.001

^{a} In aerated DMSO. ^{b} In aerated PBS/DMSO (9:1, v/v). ^{c} In deaerated PBS/DMSO (9:1, v/v). ^{d} The luminescence lifetime is 270 ns in the presence of 100 μM of $CoCl_2$. ^{e} Not determined.

Fig. S1 Percentage of surviving cells after exposure to complexes **1** – **4** at 37 °C for 24 h.

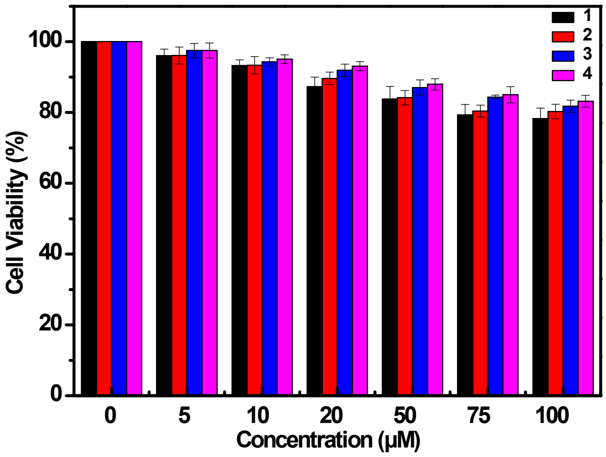

Fig. S2 Laser-scanning luminescence confocal microscopy images of HeLa cells incubated with complex **3** for 0.5 – 6 h and co-stained with CellMask.

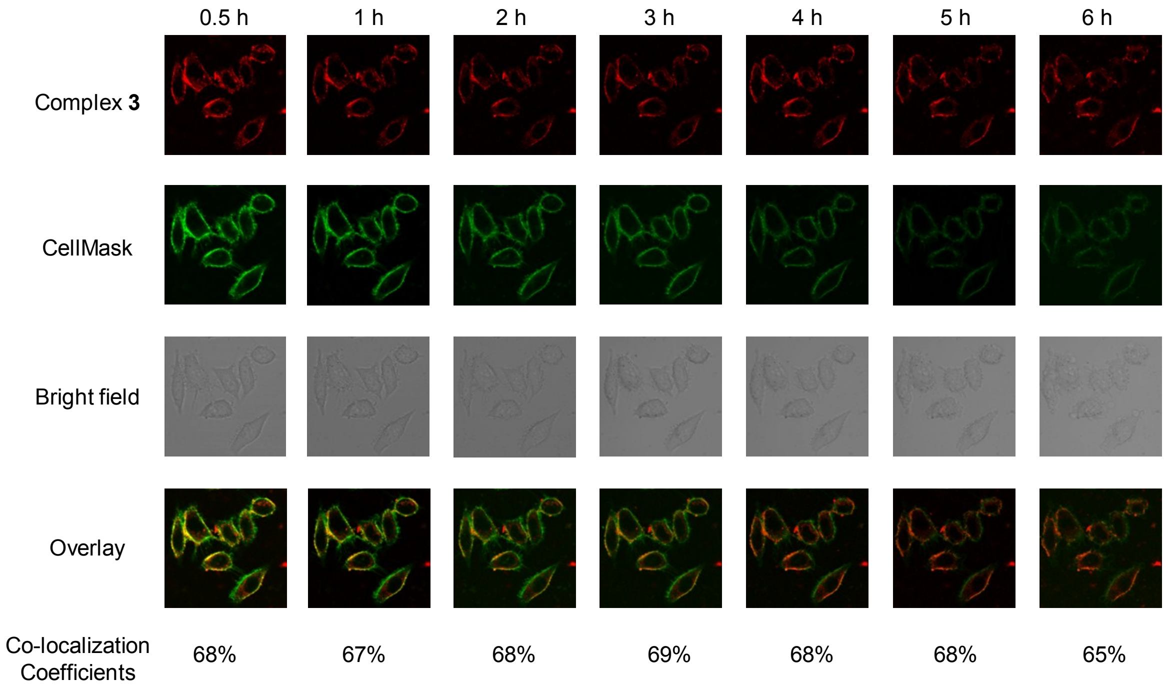

Fig. S3 Luminescence spectra of complex **3** in PBS/DMSO(9:1, v/v) under an atmosphere of 0 to 100% O_2 at 298 K and corresponding Stern-Volmer plot.

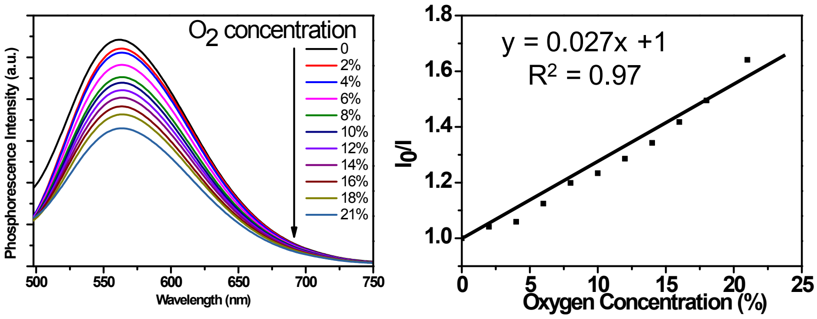

Fig. S4 Laser-scanning luminescence confocal microscopy and photoluminescence lifetime imaging microscopy images of living HeLa cells incubated with complex **3** (5 μM, 20 min, 37 °C) before and after bubbling a gas mixture of 5% O_2 and 95% N_2 into the culture medium for 5 – 60 min.

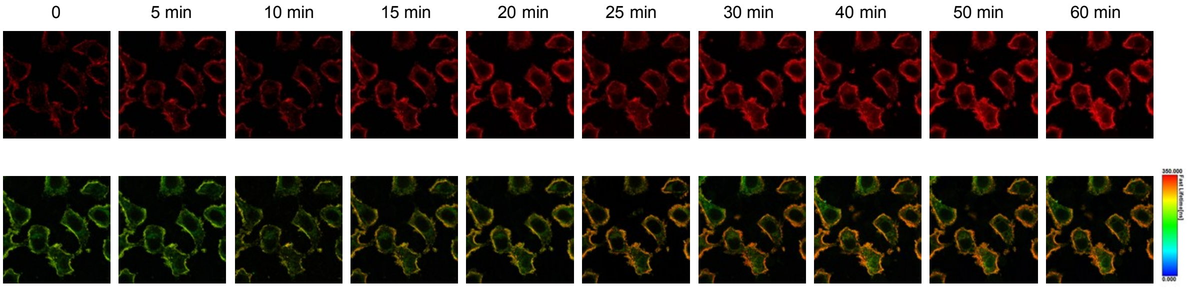

Fig. S5 Phosphorescence spectra of complex **3a** (10 μM) in CH_3OH in the presence of 0 – 50 μM of hypochlorite at 298 K. The insert shows the plot of phosphorescence intensity at 575 nm vs the concentration of hypochlorite.

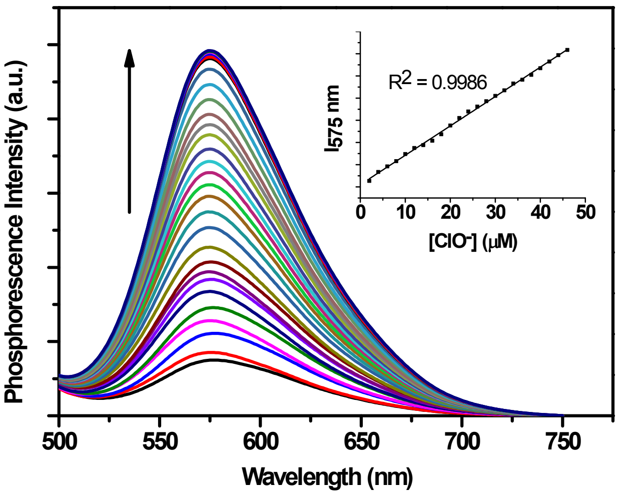

Fig. S6 Phosphorescence intensities of complex **3a** (10 μM) in the presence of different RONS (200 μM) or hypochlorite (50 μM) at 298 K.

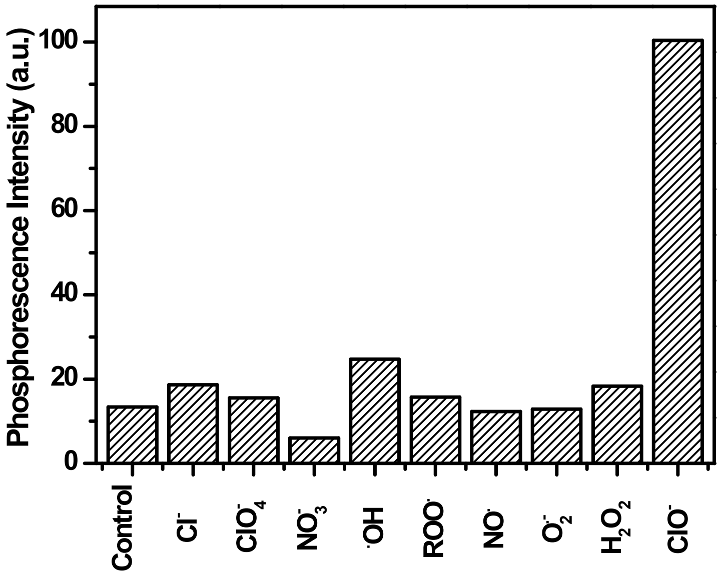

Fig. S7 Percentage of surviving cells after exposure to complexes **3a** at 37 °C for 24 h.

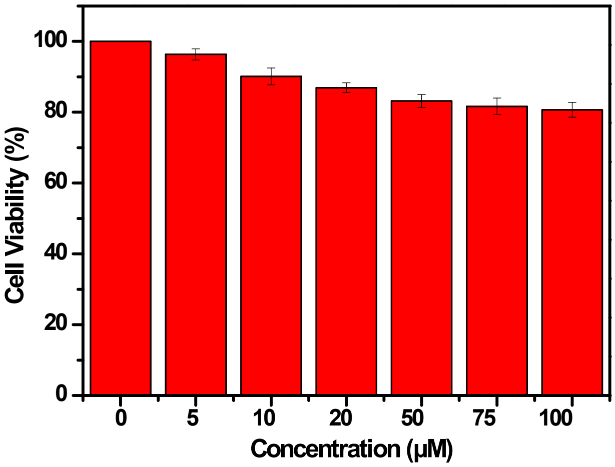

Fig. S8 Laser-scanning luminescence confocal microscopy iamges of HeLa cells incubated with complex **3a** (5 μM, 20 min, 37 °C)**.**

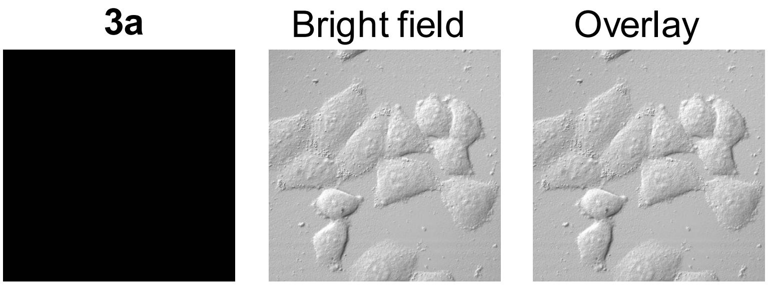

Fig. S9 Laser-scanning luminescence confocal microscopy images of the hypochlorite- and elesclomol-treated cells incubated with complex **3** (5 μM, 20 min, 37 °C).

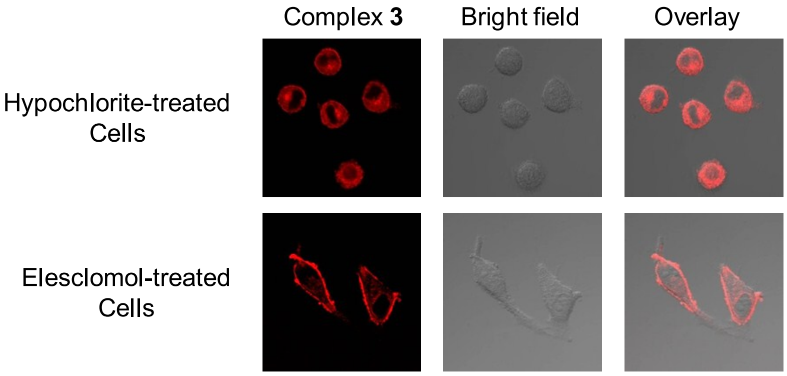

Supplement: Supplementary file 1 [file SC-009-C8SC02984A-s001.pdf]
